# Sustainable Systems Engineering: Opportunities and Challenges

**DOI:** 10.1007/s12599-022-00784-6

**Published:** 2023-01-12

**Authors:** Wil M. P. van der Aalst, Oliver Hinz, Christof Weinhardt

**Affiliations:** 1 Lehrstuhl für Informatik 9, RWTH Aachen, Ahornstr. 55, 52056 Aachen, Germany; 2 Faculty of Economics and Business Administration, Goethe University Frankfurt, Theodor-W.-Adorno-Platz 4, 60323 Frankfurt Am Main, Germany; 3 Institute of Information Systems and Marketing (IISM), Karlsruhe Institute of Technology (KIT), Kaiserstr. 89-93, 76133 Karlsruhe, Germany

## Introduction

**Fig. 1:**
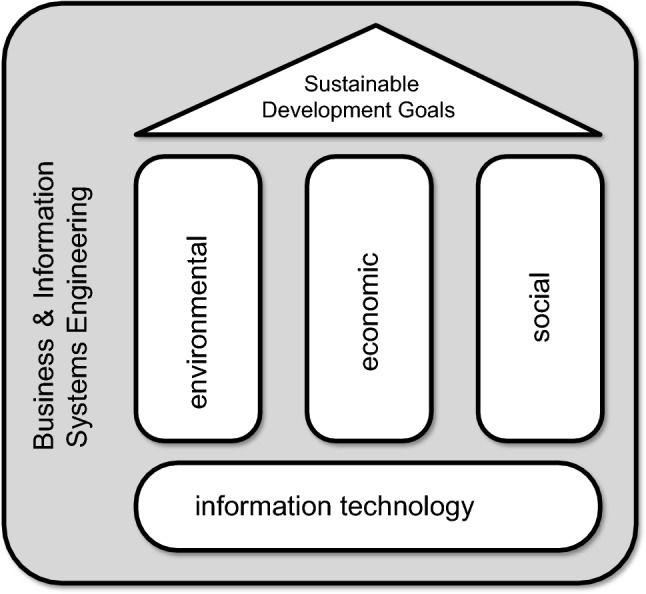
The Sustainable Development Goals (SDGs) defined by the UN cover three dimensions: environmental, economic, and social. Here, we focus on the first dimension and the role of Business and Information Systems Engineering (BISE)

The 2030 Agenda for Sustainable Development, adopted by all United Nations (UN) member states, aims to ensure that living conditions and required resources meet current human needs without undermining the integrity and stability of the natural system in the long run (United Nations 2015). The UN defined seventeen *Sustainable Development Goals* (SDGs). These SDGs aim to end poverty, improve health and education, reduce inequality, and stimulate economic growth while addressing climate change and ensuring ecological integrity (United Nations 2015). Clearly, Business and Information Systems Engineering (BISE) plays a crucial role in realizing these goals. There seems to be a consensus that sustainability has at least three dimensions (also called pillars): (1) *environmental*, (2) *economic*, and (3) *social*. Although all three dimensions are important and interrelated, we would like to focus on the *environmental dimension* and how this relates to BISE (Fig. 1).

Until now, there has been a strong correlation between human welfare and ecological footprints. In developed countries scoring high on the human development index, the ecological footprint per capita is much higher than in less developed countries. However, people realize increasingly that in the long run this will lead to global disasters ranging from global warming and rising sea levels to crop failures, undernutrition, and economic instability. Energy and food shortages due to the Ukraine war and broken supply chains due to the Covid-19 pandemic signaled the need for change. *Business and Information Systems Engineering* (BISE) as a community will need to play an essential role in this. Artificial Intelligence (AI), Machine Learning (ML), and various forms of automation (e.g., Robotic Process Automation) will continue to transform the world. Therefore, researchers and legislators are concerned with the ethics of these technologies and trying to address problems such as the lack of explainability of black-box algorithms (Bauer et al. 2021) and infringements of citizens’ rights (e.g., privacy violations and biases). However, when it comes to climate change, we are facing even bigger challenges. Therefore, topics such as circularity deserve more attention.

With this editorial, we would like to stimulate more research devoted to “*BISE for sustainability*” and the “*sustainability of BISE*”. The first focuses on the development of information systems to improve the sustainability of existing products and systems. The second focuses on the direct implications of information processing on the environment, e.g., the energy use of large server farms. According to the New York Times, creating bitcoins to spend or trade consumes around 91 terawatt-hours of electricity annually, more than the whole energy usage of a country like Finland (Huang et al. 2021). The training of neural networks is also becoming an increasing factor in energy consumption. Training the state-of-the-art language generation model GPT-3 took weeks and costed millions of dollars. Wolff Anthony et al. (2020) show that training the GPT-3 model required 190,000 kilowatt-hours of electricity, thus producing the same amount of carbon emissions as driving a car over a distance roughly equivalent to a trip to the moon and back.

The remainder is organized as follows. First, we focus on the role of information systems in enabling circularity (e.g., supporting reduce, reuse, and recycle decisions). Second, we show that it is not so easy to define sustainability and use the so-called “resource value retention options” (also known as R-imperatives) to identify opportunities for using existing technologies (e.g., process mining). Finally, we reflect on the sustainability of information technology itself. The overarching goal of this editorial is to encourage IS academics and practitioners to think about what information systems are needed to “meet the needs of the present generation without compromising the ability of future generations to meet their own needs” (United Nations 2015).

## From 3 to 10R

True sustainability necessitates the realization of a *Circular Economy* (CE). Several metrics have been developed to quantify the circularity of a system (Corona et al. 2019). For example, the fraction of a product that comes from used products (known as the *new product-level circularity metric*), or the share of recycled materials as part of the total material inputs into the global economy (known as the *global circularity metric*). In addition, several CE assessment frameworks have been developed (Vermeulen et al. 2018). Life Cycle Assessment (LCA) considers the environmental impacts of products or services during their entire life cycle. LCA includes upstream (e.g., suppliers) and downstream (e.g., waste management) and covers all inputs taken from the environment (e.g., crude oil) as well as emissions and waste (e.g., carbon dioxide). It is also possible to use more economic-oriented input–output models; however, quantification is difficult and multi-faceted (Osztovits et al. 2018). This also applies to methods to improve sustainability. In the context of CE, these are referred to as *resource value retention options* or *R-imperatives* (Vermeulen et al. 2018). The classical course-grained three R’s of waste management (3R) refer to (1) *reduce*, (2) *reuse*, and (3) *recycle*. Reduce refers to avoiding products or services or looking for alternatives. This ranges from printing on both sides to reduce paper usage or having an online meeting rather than a physical meeting. Reuse refers to using existing products again for the same or a different purpose. Recycling means that the product is transformed into a raw material used to create a new product. The original 3R classification of resource value retention options has been extended in different directions, sometimes adding new elements and sometimes refining existing classes.

Here we adopt the 10R classification introduced in Vermeulen et al. (2018) based on an extensive literature study. The ten resource value retention options can be described as follows:

- *R0 – Refuse* refers to not wanting to have the product in the first place, e.g., not buying a product or refusing complimentary items such as shopping bags or packaging material.
- *R1 – Reduce* refers to using products less frequently or using them with more care, such that they can be used longer, e.g., using a car less frequently or sharing a car.
- *R2 – Resell/reuse* refers to the usage by a second, third, fourth, etc. customer using the product without any modifications.
- *R3 – Repair* refers to extending the lifetime of the product without making changes to its function. Next to unplanned repairs, this includes maintenance to extend the lifetime.
- *R4 – Refurbish* refers to replacing components of a product without completely disassembling it. Components may be replaced due to wear or to meet new requirements (e.g., upgrading the communication unit from 3 to 5G).
- *R5 – Remanufacture* refers to completely disassembling the product and checking, cleaning, repairing, or replacing components. Consider, for example, remanufactured toner cartridges. The returned empty cartridges are disassembled, and components are cleaned and evaluated. Some parts may be replaced, and the cartridge is refilled with new toner.
- *R6 – Repurpose* refers to the usage of a product for a different purpose than initially intended. This includes transforming glass bottles into drinking mugs and using car batteries to store solar energy in homes.
- *R7 – Recycle materials* refers to the reuse of the material without retaining the original structure or function. This may include shredding or melting. Often there is a degradation of quality compared to virgin (i.e., unused raw) material.
- *R8 – Recover energy* refers to capturing energy from waste or using by-products from production processes.
- *R9 – Remine* refers to scrapping valuable materials and items from landfills, e.g., extracting rare metals like chromium and vanadium from waste.

**Fig. 2:**
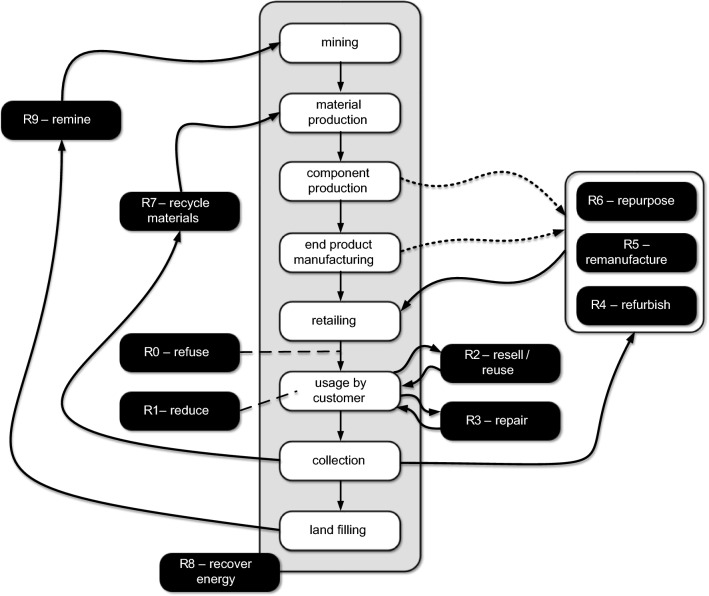
Illustration of the 10R resource value retention options. The middle part shows the traditional product flow without considering sustainability aspects. R0–R10 correspond to the R-imperatives supporting circularity

Figure 2 shows ten retention options and links these to the traditional flow from raw materials to waste. The figure is inspired by the more fine-grained literature study presented in Vermeulen et al. (2018), where the authors analyzed 69 papers discussing resource value retention options. Note that some of the “R”-s are partly overlapping or not clearly separable. Therefore, R4 (Refurbish), R5 (Remanufacture), and R6 (Repurpose) are grouped. R4, R5, and R6 may also be combined with virgin materials or components (see dashed lines). R8 (Recover energy) cannot be pinned down to a specific phase in the traditional flow and is, therefore, not connected to the rest.

Despite its imperfections, Fig. 2 may help to identify opportunities where information systems can be of help. For example, to create systems that better identify components and products, their usage, and composition. A digital passport showing origin, materials, and usage history helps to make better-informed decisions. Industry 4.0 initiatives will need to take this into account (Jamwal et al. 2021). Sustainability considerations will also lead to new business models (Boll et al. 2022). For example, there will be a shift from *owning* products to *using* products (pay-per-use or pay-per-month). Consider, for example, the baggage handling systems at airports where the airport pays per suitcase checked in, and the system is fully maintained by the manufacturer. The recyclable Cyclon running shoes by On AG provide another example. The shoes are made of beans, and using a subscription model each customer gets new, freshly recycled shoes every six months.

## Just Another KPI?

Figure 2 illustrates that to improve sustainability, many decisions need to be taken at different points in time. For example, how to design products that will be easier to repair, refurbish, or recycle later? How to produce products while recovering energy? How to optimize reverse logistics? Clearly, information systems need to play an important role here.

As an example, consider the use of *process mining* to improve sustainability (Hinish et al. 2022). Process mining tools such as the Celonis EMS support the extraction of event data from existing information systems in order to discover, analyze, predict, and improve operational processes. After extracting event data, the real processes can be discovered, providing end-to-end transparency (Van der Aalst 2016). These discovered processes are often very different from what stakeholders expect. Execution gaps related to unnecessary rework, duplicate payments/shipments, and unbalanced workloads can be identified. By using conformance checking, it is possible to detect undesired behaviors and take action to address these. Many execution gaps have environmental consequences because they lead to more waste, wear, or energy conception. Process efficiency and sustainability often go hand-in-hand. Reducing inventory in warehouses and waste in production saves money but often also reduces the ecological footprint of these activities.

From a technological point of view, one can consider “sustainability” as just another Key Performance Indicator (KPI) next to profit margin, average order fulfillment time, customer churn rate, conversion rate, etc. However, it is far from trivial to define sustainability KPIs. Even when considering only emission factors for the seven greenhouse gases covered by the Kyoto Protocol (i.e., carbon dioxide, methane, nitrous oxide, hydrofluorocarbons, perfluorocarbons, sulfur hexafluoride, and nitrogen trifluoride), there is little consensus on how to measure them precisely. However, there are attempts to standardize this. For example, the *GHG Protocol Corporate Accounting and Reporting Standard* provides requirements and guidance for companies and other organizations preparing a corporate-level GHG emissions inventory. Companies like Ecovadis and Climatiq provide software to compute the carbon emissions of activities and companies.

**Fig. 3:**
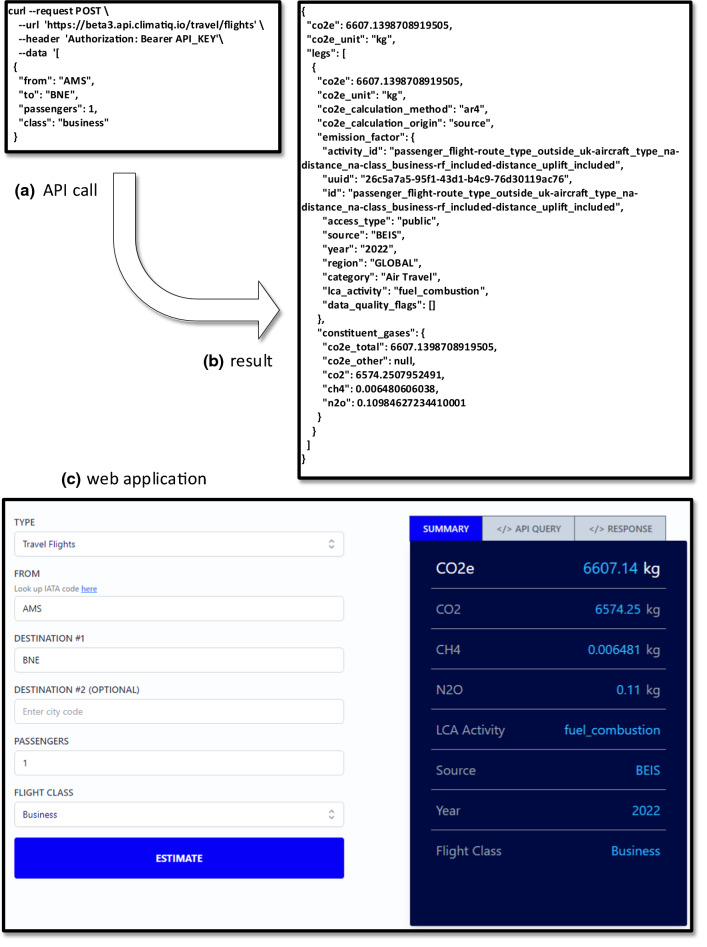
Example result of an API call asking for emission estimates of a flight from Amsterdam to Brisbane. The top part shows **a** the API call, and **b** the result is returned in JSON format. The bottom part **c** shows a small web application using this, but the same API can be embedded in any decision-making tool using 40.000 + emission factors

Figure 3 shows the *Climatiq Carbon Emissions Calculation API* in action (Climatiq 2022). A REST API can be used to pose questions about emissions. The Climatiq database contains information about over 40.000 emission factors, distributed over dozens of categories, including accommodation, air freight, air travel, cloud computing, rail freight, manufacturing, real estate, etc. Figure 3 shows an API call asking about the emission factors for a flight from Amsterdam Airport Schiphol (AMS) to Brisbane Airport (BNE) in business class. The response is based on a computation using open data and selected LCA standards. As shown, such a trip emits 6574 kg of carbon dioxide, 6.5 g of methane, and 110 g of nitrous oxide, summing up to 6607 kg of carbon dioxide equivalents. Process mining tools such as Celonis already use the APIs provided by Ecovadis and Climatiq to compute carbon footprints of processes and activities and support selecting a supplier or mode of transport. However, it should be noted that these computations are based on averages (e.g., depending on the weather, a flight may take longer and emit more carbon dioxide). Also, Fig. 2 shows that it is not easy to estimate the impact of certain decisions. When producing a product, it is not clear whether it will be recycled or remanufactured. The negative environmental impact of recycled material can be attributed to the initial product, the recycled product, or both. Figure 2 shows that the different stages and the 10R resource value retention options influence each other. This dilemma creates many interesting research problems, ranging from defining and measuring emissions to new machine learning and optimization problems.

## Computer Says No

Thus far, we have focused on “BISE for sustainability” and not on “sustainability of BISE”. However, as mentioned before, also the use of information technology has an increasing impact on the environment (Van Wynsberghe 2021). The catchphrase “Computer says no” first used in the British comedy Little Britain illustrates that it is not always good to rely on information systems (Wikipedia 2022). This also applies to situations where computations have a relatively large carbon footprint. Cryptocurrency mining and the training of sophisticated neural networks are highly energy-intensive. Moreover, in some situations, computations do not make any sense. For example, when having a lot of homogeneous data, it may be sufficient to use heuristics or sampled data. There are also situations where machine learning is infeasible and will lead to overfitting models. However, most tools and algorithms always produce a result even when it is clear that the outcome is either not reliable or is already known with enough accuracy (e.g., through caching results). Deep learning algorithms always produce a result when given enough time and resources. However, sometimes it may be better that the algorithm says “No” and refuses to compute something infeasible or unnecessary.

## Call to Action

There have been several papers that advocate a more active role of BISE researchers when it comes to sustainability (Hoffmann and Pfeiffer 2022; Loos et al. 2011; Schmidt 2009; Zeiss et al. 2021). Here we focused on the environmental dimension of sustainability. However, sustainability is also related to social and economic aspects. In Mihale-Wilson et al. (2022), we emphasized the need for corporate digital responsibility in a world where sophisticated and complex digital products and service networks create novel ethical, legal, and social challenges. Indeed, sustainability poses many grand challenges requiring collaboration across research disciplines but with a clear role for BISE. As Robert Swan said, “The greatest threat to our planet is the belief that someone else will save it”, therefore we hope this article will trigger new research ideas and solution directions.

## References

[CR1] Bauer K, Hinz O, van der Aalst W, Weinhardt C (2021). Expl(AI)n it to me – explainable AI and information systems research. Bus Inf Syst Eng.

[CR2] Boll S, Schnell M, et al (2022) Mit Künstlicher Intelligenz zu nachhaltigen Geschäftsmodellen. Whitepaper Plattform Lernende Systeme, München. 10.48669/pls_2022-1

[CR3] Climatiq (2022) Climatiq carbon emissions calculation API. https://api.climatiq.io/. Accessed 5 Dec 2022

[CR4] Corona B, Shen L, Reike D, Carreón JR, Worrell E (2019). Towards sustainable development through the circular economy – a review and critical assessment on current circularity metrics. Resour Conserv Recycl.

[CR5] Hinish S, Reinkemeyer L, Butner K, Nakladal J, Wright J (2022) The resilient digital supply chain: how intelligent workflows balance efficiency and sustainability. Research Insights. IBM Institute for Business Value. https://www.ibm.com/thought-leadership/institute-business-value/report/digital-supply-chain

[CR6] Hoffmann G, Pfeiffer J (2022). Gameful learning for a more sustainable world. Bus Inf Syst Eng.

[CR7] Huang J, O’Neill C, Tabuchi H (2021) Bitcoin uses more electricity than many countries. How is that possible? The New York Times, 3 Sept 2021. https://www.nytimes.com/interactive/2021/09/03/climate/bitcoin-carbon-footprint-electricity.html

[CR8] Jamwal A, Agrawal R, Sharma M, Kumar V, Kumar S (2021). Developing a sustainability framework for Industry 4.0. Procedia CIRP.

[CR9] Loos P, Nebel W, Marx Gómez J (2011). Green IT: a matter of business and information systems engineering?. Bus Inf Syst Eng.

[CR10] Mihale-Wilson C, Hinz O, van der Aalst W (2022). Corporate digital responsibility. Bus Inf Syst Eng.

[CR11] Osztovits A, Bagyinka F, Nádasy B, Pataki F, Perger J (2018) Closing the loop – the circular economy, what it means and what it can do for you. Whitepaper PriceWaterhouseCoopers. https://www.pwc.com/hu/en/kiadvanyok/assets/pdf/Closing-the-loop-the-circular-economy.pdf

[CR12] Schmidt NH, Erek K, Kolbe L (2009). Sustainable information systems management. Bus Inf Syst Eng.

[CR13] United Nations (2015) Transforming our world: the 2030 agenda for sustainable development (A/RES/70/1). https://sdgs.un.org/2030agenda

[CR14] van der Aalst W (2016). Process mining: data science in action.

[CR15] van Wynsberghe A (2021). Sustainable AI: AI for sustainability and the sustainability of AI. AI Ethics.

[CR16] Vermeulen WJV, Reike D, Witjes S (2018). The circular economy: new or refurbished as CE 3.0? – Exploring controversies in the conceptualization of the circular economy through a focus on history and resource value retention options. Resour Conserv Recycl.

[CR17] Wikipedia (2022) Computer says no. Wikipedia. https://en.wikipedia.org/wiki/Computer_says_no. Accessed 1 Dec 2022

[CR18] Wolff Anthony LF, Kanding B, Selvan R (2020) Carbontracker: tracking and predicting the carbon footprint of training deep learning models. ICML Workshop on Challenges in Deploying and monitoring Machine Learning Systems. https://arxiv.org/abs/2007.03051

[CR19] Zeiss R, Ixmeier A, Recker J, Kranz J (2021). Mobilising information systems scholarship for a circular economy: review, synthesis, and directions for future research. Inf Syst J.

